# Rates and risk factors for preterm birth and low birthweight in the global network sites in six low- and low middle-income countries

**DOI:** 10.1186/s12978-020-01029-z

**Published:** 2020-12-17

**Authors:** Yamini V. Pusdekar, Archana B. Patel, Kunal G. Kurhe, Savita R. Bhargav, Vanessa Thorsten, Ana Garces, Robert L. Goldenberg, Shivaprasad S. Goudar, Sarah Saleem, Fabian Esamai, Elwyn Chomba, Melissa Bauserman, Carl L. Bose, Edward A. Liechty, Nancy F. Krebs, Richard J. Derman, Waldemar A. Carlo, Marion Koso-Thomas, Tracy L. Nolen, Elizabeth M. McClure, Patricia L. Hibberd

**Affiliations:** 1 Lata Medical Research Foundation, Nagpur, India; 2 Datta Meghe Institute of Medical Sciences, Wardha, India; 3 RTI International, Durham, NC USA; 4 Instituto de Nutrición de Centroamérica y Panamá, Guatemala City, Guatemala; 5 Department of Obstetrics and Gynecology, Columbia University School of Medicine, New York, NY USA; 6 KLE Academy Higher Education and Research J N Medical College , Belagavi, Karnataka India; 7 Aga Khan University, Karachi, Pakistan; 8 Moi University School of Medicine, Eldoret, Kenya; 9 University Teaching Hospital, Lusaka, Zambia; 10 University of North Carolina at Chapel Hill, Chapel Hill, NC USA; 11 Indiana School of Medicine, University of Indiana, Indianapolis, IN USA; 12 University of Colorado School of Medicine, Denver, CO USA; 13 Thomas Jefferson University, Philadelphia, USA; 14 University of Alabama at Birmingham, Birmingham, AL USA; 15 Eunice Kennedy Shriver National Institute of Child Health and Human Development, Bethesda, MD USA; 16 School of Public Health, Boston University, Boston, MA USA

**Keywords:** Preterm, Low birth weight, Low and middle-income countries, Global network, Risk factors, India, Africa, Guatemala

## Abstract

**Background:**

Preterm birth continues to be a major public health problem contributing to 75% of the neonatal mortality worldwide. Low birth weight (LBW) is an important but imperfect surrogate for prematurity when accurate assessment of gestational age is not possible. While there is overlap between preterm birth and LBW newborns, those that are both premature and LBW are at the highest risk of adverse neonatal outcomes. Understanding the epidemiology of preterm birth and LBW is important for prevention and improved care for at risk newborns, but in many countries, data are sparse and incomplete.

**Methods:**

We conducted data analyses using the Global Network’s (GN) population-based registry of pregnant women and their babies in rural communities in six low- and middle-income countries (Democratic Republic of Congo, Kenya, Zambia, Guatemala, India and Pakistan). We analyzed data from January 2014 to December 2018. Trained study staff enrolled all pregnant women in the study catchment area as early as possible during pregnancy and conducted follow-up visits shortly after delivery and at 42 days after delivery. We analyzed the rates of preterm birth, LBW and the combination of preterm birth and LBW and studied risk factors associated with these outcomes across the GN sites.

**Results:**

A total of 272,192 live births were included in the analysis. The overall preterm birth rate was 12.6% (ranging from 8.6% in Belagavi, India to 21.8% in the Pakistani site). The overall LBW rate was 13.6% (ranging from 2.7% in the Kenyan site to 21.4% in the Pakistani site). The overall rate of both preterm birth and LBW was 5.5% (ranging from 1.2% in the Kenyan site to 11.0% in the Pakistani site). Risk factors associated with preterm birth, LBW and the combination were similar across sites and included nulliparity [RR − 1.27 (95% CI 1.21–1.33)], maternal age under 20 [RR 1.41 (95% CI 1.32–1.49)] years, severe antenatal hemorrhage [RR 5.18 95% CI 4.44–6.04)], hypertensive disorders [RR 2.74 (95% CI − 1.21–1.33], and 1–3 antenatal visits versus four or more [RR 1.68 (95% CI 1.55–1.83)].

**Conclusions:**

Preterm birth, LBW and their combination continue to be common public health problems at some of the GN sites, particularly among young, nulliparous women who have received limited antenatal care services.

*Trial registration* The identifier of the Maternal and Newborn Health Registry at ClinicalTrials.gov is NCT01073475.Trial registration: The identifier of the Maternal and Newborn Health Registry at ClinicalTrials.gov is NCT01073475.

## Background

Addressing the global burden of preterm birth (before 37 weeks of pregnancy) is critical to reducing neonatal and childhood mortality and to achieving Sustainable Development Goal #3—to ensure healthy lives and to promote well-being for all at all ages [1]. The World Health Organization (WHO) estimates that there are 15 million preterm births every year [2]. Gestational age is more predictive of risk of neonatal and childhood mortality than low birth weight (LBW—below 2500 g), but prematurity is more difficult to ascertain accurately than birthweight. Ninety-seven percent of LBW babies are born in low and lower-middle income countries (LMIC) where estimates of gestational age are the most difficult to ascertain. Therefore, the WHO also estimates the number of babies born with LBW, currently 25 million babies annually [2]. Although prematurity is a major reason for a baby being born LBW, LBW is an imperfect surrogate for preterm birth. Term babies may also be LBW because they are growth restricted and small for gestational age (SGA) that weigh less than 10th percentile of weight for gestational age and sex, resulting in an estimated 67% overlap between preterm birth and LBW [3, 4]. Mechanisms and risk factors for preterm and for LBW babies may differ despite a substantial proportion of LBW being contributed by preterm births as LBW infants are also a result of intrauterine growth restriction [5]. LBW and/or preterm birth are important causes of neonatal mortality. In 2015, there were an estimated 1 million deaths in children under age 5 years globally attributed to prematurity [6]. Infants who are SGA have an increased risk of neonatal mortality regardless of their association with preterm birth [7]. Thirty five percent of neonatal deaths were attributed to prematurity [8] and more than 80% of neonatal deaths were in LBW babies [9].

There have been calls for improved estimates of the burden of preterm birth and LBW, particularly in countries where the data are sparse, incomplete or not population-based [8]. There is limited information on maternal factors associated with these neonatal conditions and whether and how they may occur in rural settings in LMICs. More accurate data may enable government policies and programs to more effectively target interventions to reduce preterm birth and LBW. Neonatal mortality in LBW preterm babies is higher, with more severe lifelong consequences, than for LBW and preterm babies alone and therefore accurate estimates of and risk factors for the combination are needed to improve survival of these newborns [10].

The *Eunice Kennedy Shriver* National Institute of Child Health and Human Development’s (NICHD’s) Global Network (GN), is a multi-site research network representing partnerships of U.S. and international investigators at rural and semi-urban study sites in Guatemala, India (2 sites:Nagpur and Belgaum), Pakistan, Kenya, Zambia and the Democratic Republic of the Congo. The GN Maternal and Newborn Health Registry (MNHR) has been collecting data on a population-based sample of pregnant women and their babies starting in 2008. The GN has consistently focused on improving the quality of its data [11], by focusing on obtaining accurate and standardized methods of assessing birth weight and gestational age data across all participating sites. Gestational age data has been improved over time by increased access to ultrasound dating, mostly from January 2014. Standardized training of sonographers has also been possible across the GN as ultrasounds were required for three GN studies [12–14] that drew their study participants from subjects participating in the MNHR. Here we describe and compare the rates of preterm births, LBW and a combination of preterm birth and LBW at the GN sites. We also explored and compared the maternal, delivery and infant characteristics as risk factors associated with preterm birth, LBW and both preterm birth and LBW for the GN sites.

## Methods

### Study design, setting and participants

This study is a secondary data analysis using the GN’s population-based MNHR. The details of the MNHR have been published [15]. The MNHR started in 2008 and has registered approximately 70,000 pregnant women and their babies annually in rural and semi-urban communities in the countries listed above. Each site has included 6–24 distinct geographic locations (clusters). The registry continues to prospectively identify, consent, screen and enroll pregnant women in the study communities as early as possible in their pregnancy. The enrollment target in all participating communities (clusters) is at least 95% of pregnant women. Pregnant women included in this analysis were screened, consented and enrolled in the MNHR between January 2014 and December 2018, since first trimester ultrasound for gestational age dating and quality assurance efforts to improve the accuracy of reporting of last menstrual period (LMP) were consistently adopted sites.

### Eligibility criteria

Women were excluded from the analysis if they did not consent; were lost to follow-up prior to delivery; or if the information on gestational age at birth, vital status or birth weight was missing. Women who died prior to delivery, who had a miscarriage or medical termination of pregnancy, a multiple birth or a stillbirth were also excluded from analysis.

### Data collection process, co-variates and quality control

Data on the enrolled women were collected by trained health workers at three time points: at enrolment (as early as possible in pregnancy: age, educational status, height [except in the Kenya site that did not collect maternal height until 2017], weight, maternal BMI collected on enrollment, parity, date of LMP, expected date of delivery, and hemoglobin levels [two sites that had data available for most of the study period]); at delivery (receipt of iron, vitamins or calcium and the tetanus toxoid immunization during this pregnancy; number of antenatal care (ANC)visits; antenatal ultrasound information; date of delivery; gender of the baby; birth weight; mode of delivery; maternal conditions such as obstructed labor, severe antepartum or postpartum hemorrhage, hypertensive disorders, and fetal malpresentation; neonatal status; and place of delivery); at 42 days postpartum (maternal mortality, neonatal survival, and hospitalizations of the mother or baby) [16–20].

Senior Foreign Investigators (on-site primary investigators) at all sites were trained centrally. They then trained their site’s data collectors prior to collecting study data. Data sources included medical records as well as interviews with the participating women. Data collected on paper were entered into a database at a site-based data management center and transmitted to a central data coordinating center at Research Triangle Institute (RTI), Durham, NC, USA. RTI monitored the data with monthly reports of data quality (completeness and timeliness) and edit reports to identify out of range or inconsistent data that were then addressed by the site staff as well site visits.

#### Outcomes

The primary outcomes for the study were rates of preterm birth, rates of LBW and their combination. Preterm birth rate was defined as the number of neonates delivered before 37 completed weeks of gestation per 100 live births. Gestational age was estimated from the most reliable variable using the following hierarchy of reliability (1) ultrasound, (2) date of LMP, if menses are regular, (3) neonatal examination and (4) gestational age recorded at birth. The LMP was considered reliable if the LMP date given was consistent with other information about the pregnancy (i.e., plausible date), the pregnant woman states that she has a regular menstrual cycle, seems relatively sure of the date and was not using contraception. Ultrasound examinations were conducted either as a part of a GN study wherein standardized training was provided to sonologists or as a part of routine antenatal care. However, availability of ultrasound information any time during pregnancy with information on gestational age varied across sites. Additionally, babies with birthweight between the 99th percentile at 36 week (based on country-specific growth data: DRC = 3086, Zambia = 3093, Guatemala = 2959, Belagavi, India = 2784, Pakistan = 2849, Nagpur = 2680, Kenya = 3311) and 5500 g were classified as term births [21, 22].

Low birth weight (LBW) rate was defined as the number of neonates with a birthweight less than 2,500 g per 100 live births. Birth weight was measured (not estimated) within 6 days of birth in more than 96.8% of neonates in the MNHR [15].

Preterm and LBW rate was defined as the number of neonates both preterm birth and LBW per 100 live births.

#### Ethical clearance

The Institutional Review Boards and Ethics Research Committees of all participating institutions approved the MNHR. Prior to initiation of the study, agreement to participate was obtained from the communities through sensitization meetings. Individual informed consent for study participation was required from each study participant. No monetary reimbursements were provided to study participants nor to the communities participating in the study. A Data Monitoring Committee appointed by NICHD oversaw and reviewed the study at annual meetings.

### Statistical analysis

We computed summary statistics (e.g., n and proportions) overall and by GN site for rates of preterm birth, LBW and the combination of preterm and LBW, as well as maternal and neonatal characteristics. Generalized linear models (GLM) with generalized estimating equation (GEE) working correlation structure were used to evaluate the relationship of potential factors and preterm birth, LBW and the combination of preterm birth and LBW to develop point and interval estimates of relative risk associated with these factors. GEE were used to account for the correlation of outcomes within cluster to develop appropriate confidence intervals. The risk ratios for the factors associated with preterm birth, LBW, and their combination in the overall study population and by site are represented graphically and also described using multivariable regression analyses separately for preterm birth, LBW and their combination. All analyses were conducted in SAS 9.4 (RTI Inc., Durham, NC). A two-sided p-value < 0.05 was considered to be statistically significant.

## Results

From January 2014 to December 2018, a total of 303,883 women were screened. Among these, 272,192 (89.6%) met the inclusion criteria of maternal survival through delivery and a pregnancy resulting in a singleton, live birth with gestational age and birth weight assessed.

**Fig. 1:**
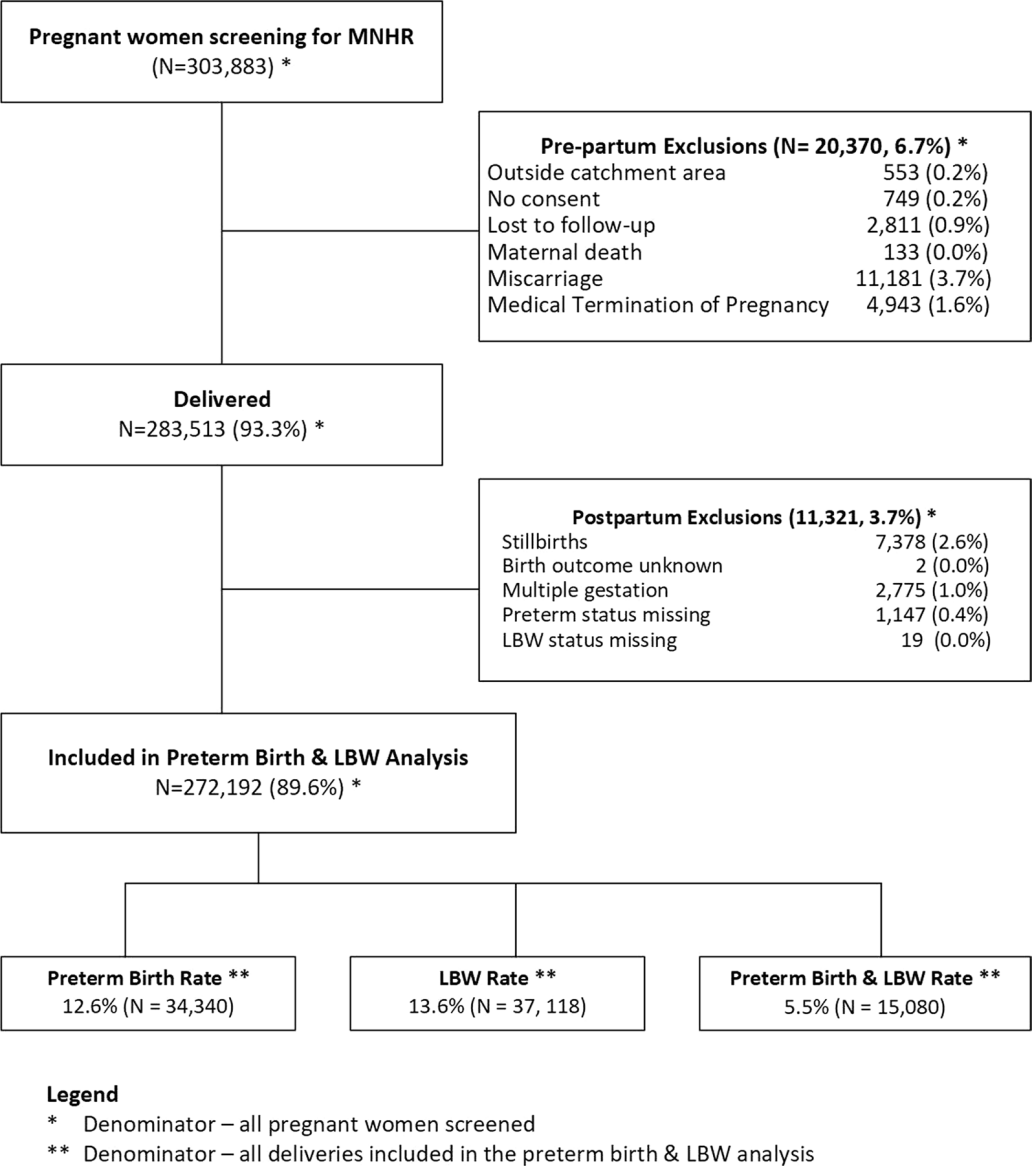
Study flow diagram

These mother-baby pairs were analyzed for rates of preterm birth, LBW and the combination of both preterm birth and LBW (Fig. 1). For this population, gestational age was based on ultrasound for 18.5% of babies, LMP for 68.1%, clinical exam for 1.6%, as collected at delivery (usually by LMP) for 6.1%, and weight for 5.7%.

### Maternal, delivery and infant characteristics of the study population

As shown in Table 1, more than 80% of the women were aged 20–35 years across all GN sites, but 19–24% of the women in the Africa sites vs. 2–12% of the women in the Asian sites were less than age 20 years of age. Overall, almost 79% had completed at least primary and secondary education, but 82% of women in the Pakistani site and 38% of women in the DRC site had no formal education.

**Table 1:** Maternal, delivery and infant characteristics by Global Network Site

Characteristic	Overall, all sites	DRC	Zambia	Kenya	Guatemala	Belagavi, India	Nagpur, India	Pakistan
Deliveries, N	272,192	29,796	33,918	36,166	52,047	40,172	41,894	38,199
Maternal age	272,094	29,767	33,906	36,110	52,047	40,172	41,894	38,198
< 20	38,388 (14.1)	5744 (19.3)	194 (24.2)	8166 (22.6)	8915 (17.1)	4684 (11.7)	981 (2.3)	1704 (4.5)
20–35	219,304 (80.6)	21,493 (72.2)	22,992 (67.8)	26,402 (73.1)	37,865 (72.8)	35,360 (88.0)	40,701 (97.2)	34,491 (90.3)
> 35	14,402 (5.3)	2530 (8.5)	2720 (8.0)	1,542 (4.3)	5267 (10.1)	128 (0.3)	212 (0.5)	2003 (5.2)
Maternal education	272,095	29,795	33,910	36,119	52,047	40,170	41,858	38,196
No formal education	58,512 (21.5)	11,278 (37.9)	2509 (7.4)	528 (1.5)	6428 (12.4)	5236 (13.0)	1296 (3.1)	31,237 (81.8)
Primary/secondary	195,792 (72.0)	18,450 (61.9)	30,903 (91.1)	32,458 (89.9)	42,076 (80.8)	31,346 (78.0)	34,140 (81.6)	6419 (16.8)
University+	17,791 (6.5)	67 (0.2)	498 (1.5)	3133 (8.7)	3543 (6.8)	3588 (8.9)	6422 (15.3)	540 (1.4)
Parity	270,083	29,795	33,915	36,124	52,046	40,170	41,867	36,166
0	84,753 (31.4)	5,598 (18.8)	10,454 (30.8)	11,737 (32.5)	15,479 (29.7)	14,585 (36.3)	20,804 (49.7)	6,096 (16.9)
1–2	113,835 (42.1)	9350 (31.4)	13,300 (39.2)	14,136 (39.1)	20,972 (40.3)	22,795 (56.7)	20,239 (48.3)	13,043 (36.1)
> 2	71,495 (26.5)	14,847 (49.8)	10,161 (30.0)	10,251 (28.4)	15,595 (30.0)	2,790 (6.9)	824 (2.0)	17,027 (47.1)
Antenatal iron/calcium/vitamins	272,136	29,790	33,918	36,166	52,044	40,166	41,854	38,198
Received any	260,290 (95.6)	28,087 (94.3)	33,847 (99.8)	35,558 (98.3)	50,117 (96.3)	39,474 (98.3)	41,751 (99.8)	31,456 (82.3)
Tetanus toxoid immunization received	272,079	29,789	33,917	36,166	51,967	40,170	41,871	38,199
Yes	230,906 (84.9)	26,108 (87.6)	30,911 (91.1)	32,884 (90.9)	39,514 (76.0)	40,117 (99.9)	41,791 (99.8)	19,581 (51.3)
At least one ANC visit	272,155	29,796	33,918	36,164	52,036	40,171	41,872	38,198
Yes	266,816 (98.0)	28,775 (96.6)	33,889 (99.9)	35,826 (99.1)	49,841 (95.8)	40,159 (100.0)	41,763 (99.7)	36,563 (95.7)
Number of ANC visits	271,910	29,758	33,916	36,138	51,961	40,166	41,798	38,173
0	5339 (2.0)	1021 (3.4)	29 (0.1)	338 (0.9)	2195 (4.2)	12 (0.0)	109 (0.3)	1635 (4.3)
1–3	102,119 (37.6)	14,790 (49.7)	17,978 (53.0)	15,044 (41.6)	16,968 (32.7)	10,642 (26.5)	5,415 (13.0)	21,282 (55.8)
4+	164,452 (60.5)	13,947 (46.9)	15,909 (46.9)	20,756 (57.4)	32,798 (63.1)	29,512 (73.5)	36,274 (86.8)	15,256 (40.0)
Obstructive labor	272,180	29,796	33,918	36,166	52,045	40,171	41,885	38,199
Yes	14,021 (5.2)	438 (1.5)	869 (2.6)	1148 (3.2)	2271 (4.4)	3433 (8.5)	468 (8.3)	2394 (6.3)
Severe antepartum hemorrhage	272,179	29,796	33,917	36,166	52,043	40,171	41,887	38,199
Yes	1361 (0.5)	60 (0.2)	162 (0.5)	202 (0.6)	124 (0.2)	179 (0.4)	71 (0.2)	563 (1.5)
Severe postpartum hemorrhage	270,473	29,786	33,368	35,377	52,015	40,170	41,558	38,199
Yes	3041 (1.1)	178 (0.6)	189 (0.6)	438 (1.2)	426 (0.8)	639 (1.6)	66 (0.2)	1105 (2.9)
Hypertensive disorders	272,108	29,744	33,913	36,165	52,032	40,171	41,887	38,196
Yes	5904 (2.2)	12 (0.0)	284 (0.8)	150 (0.4)	2069 (4.0)	1538 (3.8)	983 (2.3)	868 (2.3)
Fetal malpresentation	272,154	29,774	33,917	36,163	52,044	40,171	41,886	38,199
Yes	4564 (1.7)	108 (0.4)	196 (0.6)	260 (0.7)	1499 (2.9)	653 (1.6)	755 (1.8)	1093 (2.9)
Sex of the baby	272,177	29,794	33,915	36,166	52,043	40,169	41,892	38,198
Female	132,674 (48.7)	14,351 (48.2)	16,794 (49.5)	18,026 (49.8)	25,428 (48.9)	19,391 (48.3)	20,141 (48.1)	18,543 (48.5)

Overall, 31% of the women were nulliparous, ranging from 17% in the Pakistani site to 50% in the Nagpur site. During antenatal period, more than 94% of the women received at least any one of iron, vitamins or calcium in all sites, except in the Pakistani site where only 82% had received one or more of these supplements. A similar pattern was seen with administration of tetanus toxoid (76% or more in all sites were immunized except in the Pakistan site, where the rate was 51%).

Overall, 60% of the women had four or more antenatal care (ANC) visits, with the Pakistani site having only 40% of women who had at least four ANC visits. Maternal hypertensive disorders were reported to be more common in the Asian and Guatemalan sites and lower in the African sites.

Severe antepartum hemorrhage was less than 0.6% in all sites except the Pakistani site (1.5%) and fetal malpresentation was 0.7% or less in the African sites, but between 1.6% and 2.9% in the Asian and Guatemalan sites.

### Rates of preterm birth, LBW and preterm birth and LBW

**Fig. 2:**
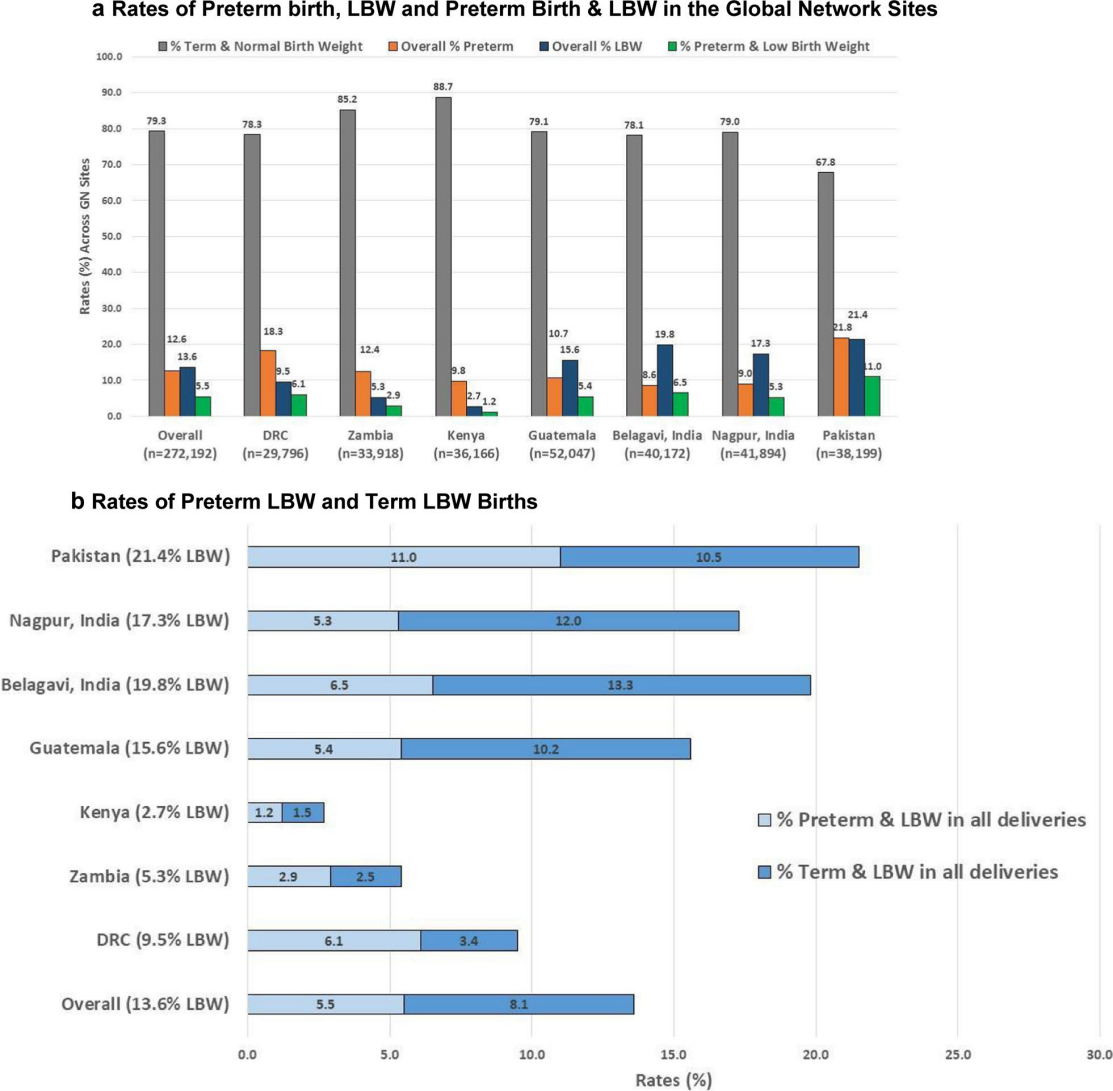
**a** Rates of Preterm birth, LBW and preterm birth and LBW in the Global Network Sites. **b** Rates of preterm LBW and term LBW births. Total % exceeds 100% because the combination of Preterm and LBW is also included in the overall Preterm and LBW rates. Normal birth weight defined as 2500 g or more

Figure 2a shows the rates of preterm birth, LBW, the combination of preterm birth and LBW as well as term, normal birth weight live births. The overall rate of preterm birth across the GN sites was 12.6%. As shown in Fig. 2a, among the Asian sites, preterm birth rates were the highest in the Pakistani site (21.8%) and lowest for the Belagavi site (8.6%). Among the African sites, DRC had the highest preterm birth rate of 18.3%. The overall LBW rate was 13.6%, in the African sites ranging from 2.7% (Kenyan site) to 9.5% (DRC site), and in the Asian sites from 17.3% (Nagpur site) to 21.4% (Pakistani site). Figure 2a also shows the rates of babies with a combination of preterm birth and LBW at 5.5%. Of note, the very low birth weight rate (those weighing < 1500 gm), was observed in 0.6% of the births overall, ranging from 0.2% in the Kenyan site to 1.1% in the Pakistani site.

Figure 2b displays the variations across sites in the proportions of LBW babies who were preterm vs. term with the Indian sites and the Guatemalan site having higher percent of term LBW babies.

### Risk factors associated with preterm birth, LBW and preterm birth and LBW

#### Risk factors associated with preterm Birth

**Fig. 3:**
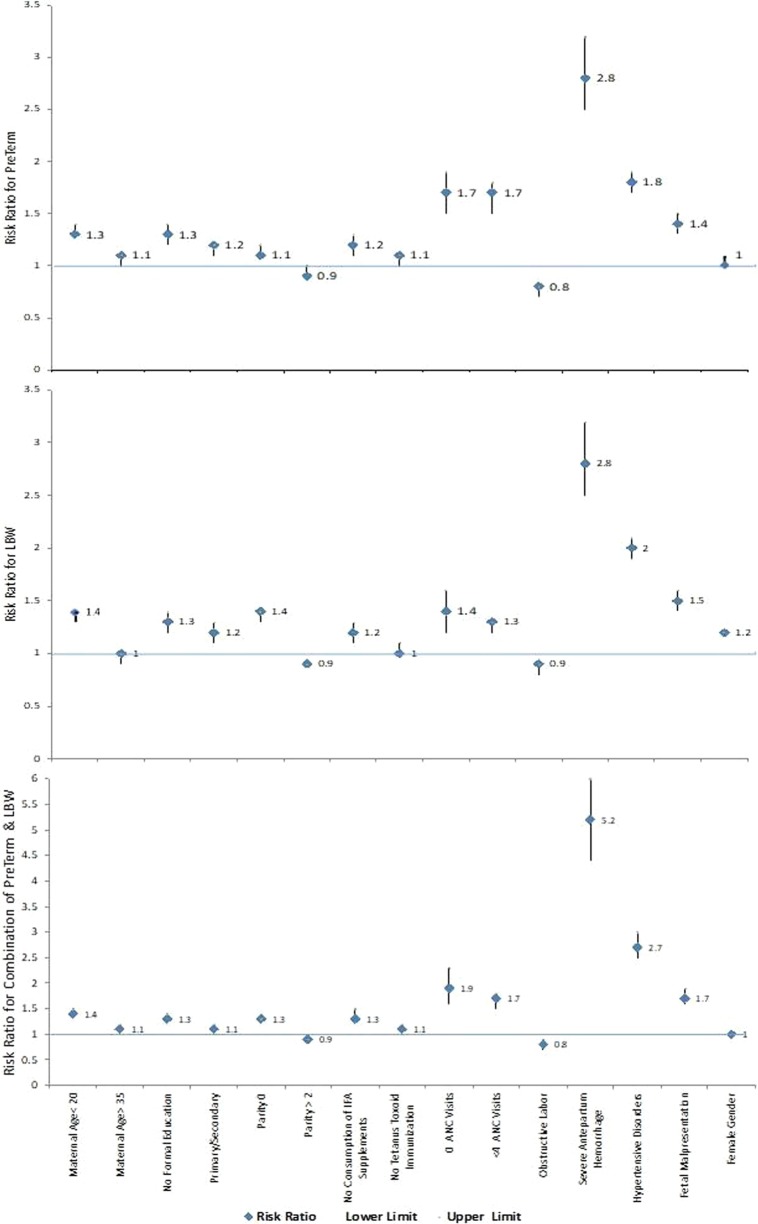
Overall risk ratios for preterm birth, LBW and preterm birth and LBW

As shown in Fig. 3, factors associated with increased risk of preterm birth in most (at least 4 of the 7 sites) GN sites included younger maternal age < 20 years, no formal education, no receipt of antenatal iron, calcium or vitamins, less than four ANC visits, severe antepartum hemorrhage, maternal hypertensive disorders, and fetal malpresentation. Of note, female gender and nulliparity was associated with preterm births at the African sites but not in the Asian sites or the Guatemalan site. Obstructed labor was associated with a decreased risk for preterm birth at most sites (see Table 2).

**Table 2:** Maternal, delivery and infant characteristics as risk factors for preterm birth—overall and site-specific risk ratios with 95% confidence intervals and p values

Characteristic	Relative risk (95% CI), p-value							
	Overall, all sites	DRC	Zambia	Kenya	Guatemala	Belagavi	Nagpur	Pakistan
Maternal age (20–35)	1.0	1.0	1.0	1.0	1.0	1.0	1.0	1.0
< 20	1.31 (1.26,1.37), < .0001	1.45 (1.30, 1.63), < .0001	1.36 (1.28, 1.44), < .0001	1.51 (1.40, 1.62), < .0001	1.30 (1.22, 1.38), < .0001	1.09 (0.99, 1.20), 0.0880	1.25 (1.07, 1.47), 0.0061	1.05 (0.93, 1.20), 0.4115
> 35	1.07 (1.02,1.12), 0.0078	1.08 (1.00, 1.18), 0.0636	1.13 (1.02, 1.25), 0.0196	1.05 (0.93, 1.19), 0.3893	1.13 (1.02, 1.24), 0.0151	2.22 (1.56, 3.16), < .0001	1.00 (0.68, 1.49), 0.9847	0.94 (0.86, 1.03), 0.1647
Maternal education (University+)	1.0	1.0	1.0	1.0	1.0	1.0	1.0	1.0
No formal education	1.32 (1.23,1.41), < .0001	2.30 (1.01, 5.25), 0.0474	1.77 (1.46, 2.14), < .0001	1.48 (1.04, 2.09), 0.0294	1.18 (0.97, 1.44), 0.0969	1.29 (1.11, 1.48), 0.0006	1.50 (1.27, 1.77), < .0001	1.13 (0.94, 1.35), 0.1895
Primary/secondary	1.17 (1.11,1.23), < .0001	2.20 (0.94, 5.14), 0.0684	1.57 (1.26, 1.95), < .0001	1.23 (1.08, 1.40), 0.0013	1.07 (0.91, 1.25), 0.4209	1.01 (0.92, 1.12), 0.7693	1.39 (1.27, 1.52), < .0001	1.01 (0.86, 1.18), 0.9492
Parity (1–2)	1.0	1.0	1.0	1.0	1.0	1.0	1.0	1.0
0	1.12 (1.07, 1.16), < .0001	1.49 (1.37, 1.62), < .0001	1.13 (1.05, 1.23), 0.0018	1.39 (1.25, 1.55), < .0001	1.00 (0.94, 1.06), 0.9133	1.10 (0.98, 1.24), 0.0944	1.05 (0.98, 1.12), 0.1429	0.98 (0.92, 1.05), 0.5708
> 2	0.93 (0.89, 0.97), 0.0002	0.95 (0.88, 1.02), 0.1329	0.95 (0.88, 1.03), 0.2046	1.11 (1.03, 1.19), 0.0045	0.94 (0.87, 1.02), 0.1227	1.07 (0.96, 1.19), 0.2446	1.31 (1.04, 1.65), 0.0243	0.87 (0.82, 0.92), < .0001
Consumption of vitamins/calcium/iron	1.0	1.0	1.0	1.0	1.0	1.0	1.0	1.0
Not consumed	1.18 (1.11, 1.25), < .0001	1.33 (1.20, 1.47), < .0001	1.24 (0.59, 2.61), 0.5700	1.46 (1.07, 2.00), 0.0178	1.10 (0.98, 1.23), 0.1168	1.78 (1.53, 2.08), < .0001	1.29 (0.86, 1.93), 0.2139	1.06 (1.03, 1.10), 0.0006
Received tetanus toxoid immunization	1.0	1.0	1.0	1.0	1.0	1.0	1.0	1.0
Did not receive	1.08 (1.02, 1.14), 0.0050	1.15 (0.99, 1.32), 0.0656	0.90 (0.75, 1.07), 0.2436	1.10 (0.96, 1.27), 0.1796	0.96 (0.88, 1.05), 0.3776	0.89 (0.30,2.64), 0.8286	1.39 (0.98,1.97), 0.0680	1.12 (1.07,1.18), < .0001
Number of ANC visits (4+)	1.0	1.0	1.0	1.0	1.0	1.0	1.0	1.0
0	1.66 (1.46, 1.88), < .0001	2.42 (1.90, 3.07), < .0001	3.97 (1.61, 9.77), 0.0027	3.28 (2.58, 4.15), < .0001	1.17 (1.02, 1.34), 0.0217	2.33 (0.89, 6.14), 0.0856	0.42 (0.17, 1.03), 0.0590	1.30 (1.14, 1.47), < .0001
1–3	1.65 (1.54, 1.78), < .0001	2.14 (1.85, 2.47), < .0001	2.48 (2.05, 3.00), < .0001	2.04 (1.85, 2.24), < .0001	1.52 (1.43, 1.63), < .0001	1.83 (1.61, 2.08), < .0001	1.30 (1.08, 1.56), 0.0050	1.16 (1.11, 1.21), < .0001
No obstructive labor	1.0	1.0	1.0	1.0	1.0	1.0	1.0	1.0
Had obstructive labor	0.77 (0.72, 0.82), < .0001	0.88 (0.77, 1.00), 0.0417	0.97 (0.77, 1.20), 0.7559	0.82 (0.68, 0.99), 0.0405	0.67 (0.53, 0.83), 0.0004	0.80 (0.65, 0.97), 0.0255	0.70 (0.60, 0.81), < .0001	0.75 (0.68, 0.83),< .0001
No severe antepartum hemorrhage	1.0	1.0	1.0	1.0	1.0	1.0	1.0	1.0
Had severe antepartum hemorrhage	2.81 (2.49, 3.18), < .0001	2.54 (1.94, 3.32),< .0001	2.89 (1.99, 4.21), < .0001	1.97 (1.50, 2.57), < .0001	4.66 (3.80, 5.72), < .0001	5.63 (4.80, 6.60), < .0001	5.19 (4.17, 6.45), < .0001	1.92 (1.76, 2.09), < .0001
No severe postpartum hemorrhage	1.0	1.0	1.0	1.0	1.0	1.0	1.0	1.0
Had severe postpartum hemorrhage	0.93 (0.84, 1.02), 0.1369	0.91 (0.65, 1.27), 0.5680	1.01 (0.69, 1.47), 0.9742	1.11 (0.93, 1.34), 0.2493	1.24 (0.93, 1.67), 0.1460	0.96 (0.78, 1.18), 0.6729	1.52 (0.83, 2.77), 0.1721	0.81 (0.74, 0.89), < .0001
No hypertensive disorders	1.0	1.0	1.0	1.0	1.0	1.0	1.0	1.0
Had hypertensive disorders	1.81 (1.68, 1.95), < .0001	2.12 (1.43, 3.15), 0.0002	1.79 (1.18, 2.72), 0.0062	1.95 (1.32, 2.87), 0.0008	1.95 (1.76, 2.17), < .0001	2.52 (2.30,2.77), < .0001	2.30 (2.05,2.57), < .0001	1.19 (1.05,1.35), 0.0075
No fetal malpresentation	1.0	1.0	1.0	1.0	1.0	1.0	1.0	1.0
Had fetal malpresentation	1.35 (1.26, 1.45), < .0001	1.31 (0.81, 2.09), 0.2679	1.53 (1.20, 1.97), 0.0007	1.11 (0.81, 1.53), 0.5165	1.44 (1.28, 1.62), < .0001	1.65 (1.26,2.15), 0.0002	0.99 (0.81,1.22), 0.9594	1.34 (1.20,1.50), < .0001
BMI, kg/m^2^ (18.5–24.9)	1.0	1.0	1.0		1.0	1.0	1.0	1.0
< 18.5	1.18 (1.15, 1.22), < .0001	1.20 (1.12, 1.28), < .0001	1.19 (1.03, 1.39), 0.0213	□	1.22 (0.98, 1.53), 0.0779	1.26 (1.16, 1.37), < .0001	1.18 (1.10, 1.26), < .0001	1.18 (1.11, 1.25), < .0001
≥ 25.0	0.80 (0.77, 0.83), < .0001	0.73 (0.63, 0.84), < .0001	0.79 (0.73, 0.85), < .0001	□	0.78 (0.73, 0.83), < .0001	1.14 (1.00, 1.29), 0.0529	0.82 (0.68, 0.98), 0.0336	0.78 (0.72, 0.85), < .0001
Hemoglobin (≥ 11 g/dL)	1.0					1.0	1.0	
10.0–10.9 g/dL (mild)	0.97 (0.90, 1.04), 0.3626	░	░	░	░	0.92 (0.82, 1.02), 0.1119	1.04 (0.97, 1.12), 0.2950	░
< 10.0 g/dL (moderate/severe)	1.10 (1.04, 1.17), 0.0013	░	░	░	░	1.04 (0.97, 1.12), 0.3015	1.20 (1.10, 1.30), < .0001	░
Sex of the baby (male)	1.0	1.0	1.0	1.0	1.0	1.0	1.0	1.0
Female	1.03 (1.01, 1.06), 0.0055	1.14 (1.08, 1.20), < .0001	1.16 (1.11, 1.21), < .0001	1.10 (1.05, 1.15), < .0001	0.94 (0.92, 0.97), < .0001	0.87 (0.82, 0.92), < .0001	1.00 (0.95, 1.06), 0.9464	1.03 (1.00, 1.07), 0.0812

□—BMI not calculated for Kenya because maternal height was not recorded before 2017

░—maternal hemoglobin not consistently collected at all GN sites except Belagavi, India and Nagpur, India

Generalized linear models were used to evaluate the relationship of potential factors and prematurity and to develop point and interval estimates of relative risk associated with these factors. Generalized estimating equations were used to account for the correlation of outcomes within cluster to develop appropriate confidence intervals. The reference group is indicated by 1.0 in the Risk Ratio column

#### Risk factors associated with LBW

Figure 3 shows that the overall risk factors for LBW were similar to the risk factors for preterm birth. As shown in Table 3, the risk factors for LBW included maternal age < 20 years, no formal education or only primary and secondary education, nulliparity, no receipt of iron, calcium or vitamins, less than four ANC visits, severe antepartum hemorrhage, maternal hypertensive disorders and fetal malpresentation. Female neonates were more likely to be LBW in all sites.

**Table 3:** Maternal, delivery and infant characteristics as risk factors for low birth weight—overall and site specific risk ratios with 95% confidence intervals and p values

Characteristic	Relative risk (95% CI), p-value							
	Overall, all sites	DRC	Zambia	Kenya	Guatemala	Belagavi	Nagpur	Pakistan
Maternal age (20–35)	1.0	1.0	1.0	1.0	1.0	1.0	1.0	1.0
< 20	1.35 (1.30, 1.40), < .0001	1.84 (1.60, 2.10), < .0001	1.67 (1.49, 1.88), < .0001	2.31 (2.10, 2.55), < .0001	1.23 (1.17, 1.28), < .0001	1.33 (1.27, 1.39), < .0001	1.23 (1.11, 1.36), < .0001	1.40 (1.27, 1.54), < .0001
> 35	1.00 (0.95, 1.05), 0.8649	1.24 (1.04, 1.49), 0.0177	0.98 (0.80, 1.21), 0.8748	0.71 (0.49, 1.02), 0.0655	1.01 (0.95, 1.07), 0.7819	1.53 (1.22, 1.91), 0.0002	1.35 (1.01, 1.79), 0.0392	0.82 (0.73, 0.91), 0.0002
Maternal education (University +)	1.0	1.0	1.0	1.0	1.0	1.0	1.0	1.0
No formal education	1.31 (1.23, 1.38), < .0001	4.56 (1.56, 13.33), 0.0056	0.76 (0.60, 0.95), 0.0158	1.21 (0.78, 1.86), 0.3961	1.16 (1.08, 1.25), < .0001	1.39 (1.24, 1.55), < .0001	1.11 (0.95, 1.30), 0.1952	1.31 (1.12, 1.53), 0.0009
Primary/secondary	1.21 (1.15, 1.27), < .0001	4.38 (1.49, 12.83), 0.0072	0.88 (0.76, 1.02), 0.0896	0.91 (0.74, 1.13), 0.4152	1.16 (1.08, 1.24), < .0001	1.21 (1.10, 1.33), < .0001	1.26 (1.16, 1.36), < .0001	1.11 (0.90, 1.36), 0.3342
Parity (1–2)	1.0	1.0	1.0	1.0	1.0	1.0	1.0	1.0
0	1.38 (1.34,1.43), < .0001	1.86 (1.69,2.05), < .0001	1.57 (1.42,1.74), < .0001	2.23 (1.93,2.59), < .0001	1.26 (1.20,1.32), < .0001	1.41 (1.34, 1.50), < .0001	1.38 (1.32, 1.45), < .0001	1.23 (1.16, 1.31), < .0001
> 2	0.90 (0.87, 0.93), < .0001	0.86 (0.79, 0.94), 0.0007	0.90 (0.83, 0.99), 0.0264	0.78 (0.67, 0.91), 0.0015	0.93 (0.87, 0.99), 0.0148	0.91 (0.85, 0.98), 0.0114	1.01 (0.86, 1.19), 0.8745	0.85 (0.80, 0.89), < .0001
Consumption of vitamins/calcium/iron	1.0	1.0	1.0	1.0	1.0	1.0	1.0	1.0
Not consumed	1.18 (1.10, 1.27), < .0001	1.35 (1.11, 1.64), 0.0030	2.20 (0.99, 4.89), 0.0521	1.71 (1.14, 2.58), 0.0100	1.16 (0.98, 1.36), 0.0779	1.47 (1.34, 1.62), < .0001	0.81 (0.60, 1.10), 0.1811	1.10 (1.03, 1.17), 0.0027
Received tetanus toxoid immunization	1.0	1.0	1.0	1.0	1.0	1.0	1.0	1.0
Did not receive	1.03 (0.97, 1.09), 0.3134	1.15 (0.90, 1.46), 0.2625	0.66 (0.48, 0.91), 0.0122	0.73 (0.52, 1.03), 0.0706	0.93 (0.87, 0.98), 0.0146	0.85 (0.53, 1.36), 0.5044	0.89 (0.61, 1.31), 0.5602	1.14 (1.07, 1.21), < .0001
Number of ANC visits (4+)	1.0	1.0	1.0	1.0	1.0	1.0	1.0	1.0
0	1.36 (1.20, 1.55), < .0001	2.48 (1.83, 3.35), < .0001	6.45 (3.12, 13.33), < .0001	2.90 (2.00, 4.20), < .0001	1.20 (0.96, 1.50), 0.1003	1.26 (0.53, 2.98), 0.5976	0.65 (0.44, 0.95), 0.0277	1.25 (1.09, 1.44), 0.0015
1–3	1.27 (1.22, 1.31), < .0001	1.88 (1.58, 2.25), < .0001	1.70 (1.39, 2.07), < .0001	1.48 (1.28, 1.70), < .0001	1.23 (1.18, 1.28), < .0001	1.33 (1.26, 1.41), < .0001	1.08 (1.00, 1.16), 0.0399	1.15 (1.08, 1.22), < .0001
No obstructive labor	1.0	1.0	1.0	1.0	1.0	1.0	1.0	1.0
Had obstructive labor	0.85 (0.80, 0.92), < .0001	1.30 (1.05, 1.60), 0.0151	1.24 (0.85, 1.81), 0.2691	1.21 (0.85, 1.72), 0.2813	0.71 (0.60, 0.84), < .0001	0.90 (0.79, 1.02), 0.1106	0.82 (0.74, 0.91), 0.0002	0.88 (0.80, 0.96), 0.0042
No severe antepartum hemorrhage	1.0	1.0	1.0	1.0	1.0	1.0	1.0	1.0
Had severe antepartum hemorrhage	2.82 (2.53, 3.15), < .0001	4.89 (3.63, 6.59), < .0001	6.56 (5.09, 8.45), < .0001	5.70 (3.93, 8.27), < .0001	3.63 (3.18, 4.14), < .0001	3.22 (2.74,3.80), < .0001	3.05 (2.43, 3.81), < .0001	1.89 (1.73, 2.07), < .0001
No severe postpartum hemorrhage	1.0	1.0	1.0	1.0	1.0	1.0	1.0	1.0
Had severe postpartum hemorrhage	1.02 (0.93, 1.12), 0.6406	1.10 (0.79, 1.53), 0.5741	1.36 (0.94, 1.97), 0.1036	2.32 (1.36, 3.97), 0.0021	1.15 (0.95, 1.38), 0.1438	0.89 (0.79, 1.01), 0.0805	1.06 (0.70, 1.60), 0.7964	0.96 (0.86, 1.07), 0.4937
No hypertensive disorders	1.0	1.0	1.0	1.0	1.0	1.0	1.0	1.0
Had hypertensive disorders	2.01 (1.88, 2.15), < .0001	1.72 (0.87, 3.40), 0.1185	3.52 (2.57, 4.82), < .0001	6.35 (3.84, 10.50), < .0001	1.99 (1.83, 2.16), < .0001	1.98 (1.86, 2.11), < .0001	1.93 (1.73, 2.14), < .0001	1.31 (1.16, 1.47), < .0001
No fetal malpresentation	1.0	1.0	1.0	1.0	1.0	1.0	1.0	1.0
Had fetal malpresentation	1.45 (1.35, 1.56), < .0001	2.43 (1.44, 4.08), 0.0008	2.25 (1.49, 3.39), 0.0001	2.13 (1.02, 4.44), 0.0432	1.40 (1.25, 1.56), < .0001	1.38 (1.16, 1.63), 0.0002	1.28 (1.09, 1.49), 0.0022	1.41 (1.28, 1.56), < .0001
BMI, kg/m^2^ (18.5–24.9)	1.0	1.0	1.0		1.0	1.0	1.0	1.0
< 18.5	1.30 (1.26, 1.34), < .0001	1.54 (1.44, 1.64), < .0001	1.45 (1.19, 1.76), 0.0002	□	1.43 (1.22, 1.66), < .0001	1.22 (1.18, 1.26), < .0001	1.24 (1.18, 1.30), < .0001	1.36 (1.31, 1.42), < .0001
≥ 25.0	0.70 (0.67, 0.73), < .0001	0.68 (0.59, 0.78), < .0001	0.57 (0.51, 0.64), < .0001	□	0.68 (0.65, 0.72), < .0001	0.80 (0.74, 0.87), < .0001	0.77 (0.69, 0.86) < .0001	0.70 (0.64, 0.76), < .0001
Hemoglobin (≥ 11 g/dL)	1.0					1.0	1.0	
10.0–10.9 g/dL (mild)	1.02 (0.98, 1.06), 0.2489	░	░	░	░	1.00 (0.96, 1.05), 0.9940	1.07 (1.01, 1.13), 0.0187	░
< 10.0 g/dL (moderate/severe)	1.12 (1.08, 1.17), < .0001	░	░	░	░	1.08 (1.03,1.14), 0.0035	1.20 (1.14, 1.26), < .0001	░
Sex of the baby (male)	1.0	1.0	1.0	1.0	1.0	1.0	1.0	1.0
Female	1.19 (1.17, 1.21), < .0001	1.24 (1.16, 1.31), < .0001	1.19 (1.09, 1.30), < .0001	1.15 (1.01, 1.31), 0.0296	1.24 (1.19, 1.28), < .0001	1.16 (1.11, 1.21), < .0001	1.20 (1.16, 1.24), < .0001	1.17 (1.12, 1.23), < .0001

□—BMI not calculated for Kenya because maternal height was not recorded before 2017

░—Maternal hemoglobin not consistently collected at all GN sites except Belagavi, India and Nagpur, India

Generalized linear models were used to evaluate the relationship of potential factors and LBW and to develop point and interval estimates of relative risk associated with these factors. Generalized estimating equations were used to account for the correlation of outcomes within cluster to develop appropriate confidence intervals. The reference group is indicated by 1.0 in the Risk Ratio column

#### Risk factors associated with both preterm birth and LBW

Figure 3 shows that the risk factors for having both a LBW and preterm birth were similar to having either a preterm or LBW birth.

However, the effect size of the antenatal care, antepartum hemorrhage and hypertensive disorder risk factors were higher than for preterm birth or LBW alone (see Table 4).

**Table 4:** Maternal, delivery and infant characteristics as risk factors for preterm birth and low birth weight—overall and site specific risk ratios with 95% confidence intervals and p values

Characteristic	Relative risk (95% CI), p-value							
	Overall, all sites	DRC	Zambia	Kenya	Guatemala	Belagavi	Nagpur	Pakistan
Maternal age (20–35)	1.0	1.0	1.0	1.0	1.0	1.0	1.0	1.0
< 20	1.41 (1.32, 1.49), < .0001	1.94 (1.61, 2.33), < .0001	1.68 (1.48, 1.90), < .0001	2.38 (2.08, 2.72), < .0001	1.32 (1.22, 1.42), < .0001	1.16 (1.06, 1.28), 0.0024	1.35 (1.11, 1.65), 0.0026	1.30 (1.15, 1.48), < .0001
> 35	1.08 (1.00, 1.17), 0.0385	1.23 (1.02, 1.48), 0.0305	1.10 (0.89, 1.35), 0.3785	0.46 (0.19, 1.07), 0.0724	1.19 (1.05, 1.35), 0.0057	2.18 (1.48, 3.22), < .0001	1.35 (0.87, 2.10), 0.1758	0.91 (0.78, 1.07), 0.2510
Maternal education (University +)	1.0	1.0	1.0	1.0	1.0	1.0	1.0	1.0
No formal education	1.28 (1.18, 1.38), < .0001		0.67 (0.54, 0.83), 0.0002	0.95 (0.49, 1.82), 0.8741	1.08 (0.89, 1.30), 0.4399	1.26 (1.10, 1.45), 0.0008	1.32 (1.06, 1.65), 0.0140	1.25 (1.04, 1.50), 0.0165
Primary/secondary	1.13 (1.06, 1.21), 0.0002		0.78 (0.64, 0.96), 0.0201	0.93 (0.64, 1.35), 0.6876	1.11 (0.96, 1.27), 0.1620	0.98 (0.88, 1.08), 0.6455	1.39 (1.23, 1.57), < .0001	1.07 (0.87, 1.32), 0.5037
Parity (1–2)	1.0	1.0	1.0	1.0	1.0	1.0	1.0	1.0
0	1.27 (1.21, 1.33), < .0001	1.95 (1.69, 2.25), < .0001	1.46 (1.36, 1.57), < .0001	2.14 (1.79, 2.57), < .0001	1.14 (1.03, 1.27), 0.0122	1.22 (1.08, 1.39), 0.0014	1.26 (1.16, 1.36), < .0001	1.12 (1.05, 1.20), 0.0010
> 2	0.90 (0.86, 0.95), < .0001	0.92 (0.84, 1.01), 0.0822	0.90 (0.80, 1.03), 0.1159	0.69 (0.58, 0.82), < .0001	0.99 (0.87, 1.12), 0.8555	1.08 (0.99, 1.18), 0.0977	1.43 (1.03, 1.99), 0.0349	0.85 (0.79, 0.91), < .0001
Consumption of vitamins/calcium/iron	1.0	1.0	1.0	1.0	1.0	1.0	1.0	1.0
Not consumed	1.30 (1.15, 1.47), < .0001	1.82 (1.44, 2.31), < .0001	2.94 (0.89, 9.77), 0.0783	1.90 (0.91, 3.95), 0.0875	1.22 (1.03, 1.45), 0.0227	2.17 (1.91, 2.46), < .0001	0.72 (0.29, 1.75), 0.4654	1.08 (1.01, 1.16), 0.0357
Received tetanus toxoid immunization	1.0	1.0	1.0	1.0	1.0	1.0	1.0	1.0
Did not receive	1.13 (1.04, 1.23), 0.0030	1.39 (1.03, 1.86), 0.0294	0.67 (0.43, 1.05), 0.0806	0.75 (0.48, 1.16), 0.1939	0.92 (0.84, 1.01), 0.0664	0.57 (0.09, 3.54), 0.5503	1.15 (0.56, 2.39), 0.7008	1.23 (1.14, 1.33), < .0001
Number of ANC visits (4 +)	1.0	1.0	1.0	1.0	1.0	1.0	1.0	1.0
0	1.88 (1.56, 2.27), < .0001	5.27 (3.76, 7.39), < .0001	15.00 (6.23, 36.10), < .0001	5.93 (4.06, 8.68), < .0001	1.29 (1.03, 1.60), 0.0257	3.14 (1.15, 8.54), 0.0252	0.35 (0.14, 0.87), 0.0234	1.32 (1.12, 1.55), 0.0007
1–3	1.68 (1.55, 1.83), < .0001	3.17 (2.57, 3.91), < .0001	3.00 (2.22, 4.06), < .0001	2.62 (2.09, 3.29), < .0001	1.59 (1.47, 1.72), < .0001	2.13 (1.87, 2.44), < .0001	1.40 (1.12, 1.75), 0.0030	1.16 (1.09, 1.24), < .0001
No obstructive labor	1.0	1.0	1.0	1.0	1.0	1.0	1.0	1.0
Had obstructive labor	0.78 (0.70, 0.87), < .0001	1.51 (1.20, 1.89), 0.0004	1.28 (0.84, 1.97), 0.2551	1.71 (1.19, 2.44), 0.0036	0.59 (0.43, 0.82), 0.0018	0.76 (0.59, 0.97), 0.0251	0.68 (0.56, 0.84), 0.0002	0.79 (0.71, 0.88), < .0001
No severe antepartum hemorrhage	1.0	1.0	1.0	1.0	1.0	1.0	1.0	1.0
Had severe antepartum hemorrhage	5.18 (4.44, 6.04), < .0001	6.63 (5.04, 8.72), < .0001	10.24 (7.15, 14.66) < .0001	10.28 (6.29, 16.77), < .0001	8.52 (6.99, 10.40), < .0001	7.40 (6.22, 8.81), < .0001	8.00 (6.16, 10.38), < .0001	2.75 (2.44, 3.11), < .0001
No severe postpartum hemorrhage	1.0	1.0	1.0	1.0	1.0	1.0	1.0	1.0
Had severe postpartum hemorrhage	1.22 (1.07, 1.40), 0.0035	1.10 (0.68, 1.77), 0.6996	1.67 (1.16, 2.42), 0.0062	3.42 (2.14, 5.46), < .0001	1.80 (1.31, 2.49), 0.0003	1.18 (0.97, 1.43), 0.0980	1.70 (0.90,3.20), 0.0996	0.96 (0.85,1.09), 0.5243
No hypertensive disorders	1.0	1.0	1.0	1.0	1.0	1.0	1.0	1.0
Had hypertensive disorders	2.74 (2.48, 3.02), < .0001	2.64 (1.17, 5.96), 0.0193	4.54 (2.82, 7.31), < .0001	10.72 (5.60, 20.51), < .0001	2.84 (2.52, 3.19), < .0001	3.09 (2.77, 3.44), < .0001	3.18 (2.80, 3.61), < .0001	1.46 (1.25, 1.72), < .0001
No fetal malpresentation	1.0	1.0	1.0	1.0	1.0	1.0	1.0	1.0
Had fetal malpresentation	1.74 (1.56, 1.93), < .0001	2.93 (1.68, 5.13), 0.0002	2.62 (1.58, 4.35), 0.0002	3.50 (1.90, 6.47), < .0001	1.80 (1.48, 2.19), < .0001	1.83 (1.42, 2.35), < .0001	1.17 (0.92, 1.50), 0.1985	1.57 (1.38, 1.78), < .0001
BMI, kg/m^2^ (18.5–24.9)	1.0	1.0	1.0		1.0	1.0	1.0	1.0
< 18.5	1.33 (1.27, 1.39), < .0001	1.40 (1.28, 1.53), < .0001	1.39 (1.20, 1.62), < .0001	□	1.42 (1.07, 1.88), 0.0139	1.26 (1.16, 1.38), < .0001	1.28 (1.15, 1.42), < .0001	1.40 (1.31, 1.50), < .0001
≥ 25.0	0.77 (0.72, 0.81), <.0001	0.68 (0.57, 0.80), < .0001	0.61 (0.54, 0.68), < .0001	□	0.73 (0.66, 0.81), < .0001	1.12 (0.95, 1.33), 0.1603	0.82 (0.63, 1.07), 0.1503	0.76 (0.69, 0.84), < .0001
Hemoglobin (≥ 11 g/dL)	1.0					1.0	1.0	
10.0–10.9 g/dL (mild)	0.96 (0.87, 1.05), 0.3580	░	░	░	░	0.93 (0.82, 1.05), 0.2182	1.04 (0.89, 1.23), 0.6142	░
< 10.0 g/dL (moderate/severe)	1.12 (1.02, 1.23), 0.0137	░	░	░	░	1.08 (0.97, 1.21), 0.1754	1.25 (1.07, 1.46), 0.0046	░
Sex of the baby (MALE)	1.0	1.0	1.0	1.0	1.0	1.0	1.0	1.0
Female	1.01 (0.98, 1.04), 0.5527	1.18 (1.09, 1.28), < .0001	1.13 (1.08, 1.19), < .0001	1.00 (0.88, 1.13), 0.9487	0.94 (0.88, 0.99), 0.0236	0.86 (0.81, 0.92), < .0001	1.00 (0.94, 1.07), 0.9333	1.07 (1.01, 1.15), 0.0338

□—BMI not calculated for Kenya because maternal height was not recorded before 2017

░—maternal hemoglobin not consistently collected at all GN sites except Belagavi, India and Nagpur, India

Generalized linear models were used to evaluate the relationship of potential factors and the combination of prematurity and LBW and to develop point and interval estimates of relative risk associated with these factors. Generalized estimating equations were used to account for the correlation of outcomes within cluster to develop appropriate confidence intervals. The reference group is indicated by 1.0 in the Risk Ratio column

#### Risk factors available at select sites

Maternal BMI was available in all sites except the Kenya site. BMI < 18.5 kg/m^2^ was a risk factor for preterm birth at all sites and LBW at nearly all sites. Maternal hemoglobin was consistently available over the study period in the two Indian sites. Mild anemia (hemoglobin 10.0–10.9 g/dL) was associated only with LBW in the Nagpur site and not associated with preterm at either of the sites. Moderate to severe anemia (< 10 g/dL) was associated with preterm birth, LBW, and the combination of preterm birth and LBW when data from the two Indian sites were combined.

## Discussion

The Global Network’s population-based estimate of the preterm birth rate was 12.6% with the highest rates in the Pakistani and the DRC sites and the lowest rates (< 10%) in the Indian and Kenyan sites. LBW rates were 13.6% but showed a different pattern with the highest rates in the Asian and Guatemalan sites and the lowest rates in the African sites. The combination of preterm birth and LBW was highest in the Pakistani site (11%) and lowest in the Kenyan site (1.2%).

Our rural and semi-urban population rates for preterm birth are similar to country-wide estimates in previously reported studies [23–28]. However, our results are higher than recent global estimates for preterm birth in 2014 (10.6%—uncertainty interval 9.0–12.0) [7]. Rates of preterm birth in the Pakistani and DRC sites were outliers among the GN sites and were associated with poor antenatal care indicators such as lack of receipt of antenatal iron, calcium and vitamins, lack of tetanus toxoid immunization and fewer than four antenatal visits [29–31]. The Asian sites had higher rates of LBW, likely because term and preterm birth weights are higher in the African sites and intrauterine growth retardation or SGA is more common in the Asian sites as observed in other studies [32–36].

Our estimates of LBW are slightly lower, but within range of recent global estimates for LBW in 2015 (14.6%—uncertainty range 12.4–17.1%) [8]. To our knowledge, there are no prior global estimates of the combination of both preterm and LBW. Risk factors for having a preterm birth and LBW baby are similar to those for each condition alone, likely because of the overlap of these outcomes. They include poor ANC indicators such as less than four ANC visits, nulliparity and maternal age under age 20 [37], severe antepartum hemorrhage and hypertensive disorders. The similarity of risk factors for preterm birth, LBW and the combination of preterm birth and LBW in the GN sites suggest an important need to monitor those women with these risk factors as it may help to improve their pregnancy outcomes.

Risk factors for prematurity, LBW and their combination varied by GN site. Therefore, the antenatal factors that are associated with these neonatal conditions may be monitored according to their region. The African sites had higher rates of preterm birth in the nulliparous women, whereas parity was not associated with preterm birth in the other GN sites. This was perhaps due to the higher proportion of women in Africa being < 20 years of age and likely to have their first pregnancy at younger ages [31, 37]. The other factor that was associated with increased risk for preterm birth in all sites was antepartum hemorrhage [38, 39]. The rates of preterm births were higher in the African sites. Whereas the rates of LBW were much higher in the non-African sites and so were the reported rates of hypertensive disorders. Hypertensive disorders are associated with SGA [40]. Another possible reason for higher rates of LBW in Asian sites could be higher rates of BMI < 18.5 kg/m^2^ and its association with LBW [41]. Moderate/severe anemia recorded anytime during pregnancy (data evaluated only for the Indian sites where enrollment in the study tended to be earlier in pregnancy than in other sites), either due to iron deficiency or other causes, increased the risk of both preterm birth and LBW. Anemia any time and especially during the rapid fetal growth during the third trimester is reported as an important factor in determining birth weight [18–20].

Another interesting observation from this study was the association of gender of the newborn with preterm birth and LBW. In African sites, female newborns were more likely to be preterm. Although reported previously the reasons for this finding are unclear [42, 43]. At all sites, the rate of LBW in female newborns was more than in males.

The strengths of our study are that it is a population-based assessment of the burden of preterm birth and LBW across different LMIC community sites and their risk factors that differ by site indicating that further exploration of association of these risk factors may help to tailor public health strategies. The GN uses uniform and rigorous criteria across sites for assessment of gestational age, increasingly based on ultrasound dating (particularly first trimester) to improve precision of estimates of preterm birth rates. The GN has also focused on accurate estimation of measured birth weight within 6 days of birth in approximately 99% of neonates, to avoid rounding errors and delayed reporting of birth weight [15]. Missing values of key variables including birthweight are minimal, particularly as a result of increased institutional deliveries.

Limitations of the study include the population under study – it is not countrywide, because it is based on access to a rural and semi-urban population. Another limitation is that while estimation of gestational age and therefore preterm birth rates are improving, they are not yet optimal.

LMP may not be a reliable indicator in this population due to recall bias and inability to remember the exact LMP by less educated individuals. Even when the LMP is known, there is a risk for potential bias as the estimation of LMP is based on the assumption of a uniform 28-day cycle with ovulation on day 14 of that cycle, both of which may not necessarily be true, especially if contraceptives have been used before conception [44–46]. There can be a lack of agreement on the definition (women estimate the LMP as the last day of the menstrual period) of LMP between the doctor and pregnant woman. This can lead to errors and discrepancies in GA assessment by LMP [47]. Ultrasound dating is also limited by the changes in the size of the fetus which is not uniform throughout the pregnancy. It does not account for growth restriction and thus can lead to misclassification of term growth restricted baby as a preterm birth [48]. The magnitude and direction of the systematic bias should be considered while using the US based estimates. Increased access to high quality ultrasound estimates early in pregnancy is likely to further improve precision of preterm birth estimates. Improved precision of gestational age will allow estimation of growth restriction at all gestational ages (SGA). Rates of SGA and its determinants at each site were not estimated in this analysis. So further research is needed to understand the risk factors of SGA and how they differ at each site, when better estimates of gestational age become available.

Additionally, the association of plausible risk factors like socio-economic status, income, history or previous preterm as well as modifiable risk factors should be accounted for in the future research.

## Conclusion

Rates of preterm births, LBWs and their combination remain high at the GN sites. Prominent risk factors that were similar across sites included nulliparity, maternal age under 20 years, less than 4 antenatal care visits, severe antenatal hemorrhage, and hypertensive disease.

While all pregnancies need care and consideration to prevent preterm births, the younger nulliparous women who may have received limited access to ANC services and so are at higher risk for preterm births need more attention to prevent prematurity and LBW.

## Data Availability

A minimal dataset for the findings described in the manuscript will be shared upon request for the same.

## Abbreviations

ANC: Antenatal care
BMI: Body mass index
DRC: Democratic Republic of Congo
g/dL: Grams per deciliter
GEE: Generalized estimating equations
GLM: Generalized linear models
GN: Global network
LBW: Low birth weight
LMICs: Lower and middle income countries
LMP: Last menstrual period
MNHR: Maternal and Newborn Health Registry
NICHD’s: National Institute for Child Health and Human Development`s
RR: Risk ratios
RTI: Research Triangle Institute
SGA: Small for gestational age
